# Morphological and Geographical Traits of the British Odonata

**DOI:** 10.3897/BDJ.2.e1041

**Published:** 2014-01-29

**Authors:** Gary D Powney, Stephen J Brooks, Louise J Barwell, Phil Bowles, Robert N L Fitt, Alyson Pavitt, Rebecca A Spriggs, Nick J B Isaac

**Affiliations:** † Centre for Ecology & Hydrology, Wallingford, United Kingdom; ‡ Department of Life Sciences, Imperial College London, Ascot, United Kingdom; § Life Sciences Department, Natural History Museum, London, United Kingdom; | School of Biology, University of Leeds, Leeds, United Kingdom; ¶ Zoology Department, University of Aberdeen, Aberdeen, United Kingdom; # Department of Plant Sciences, University of Cambridge, Cambridge, United Kingdom

**Keywords:** Odonata, trait data, body size, distribution status, habitat preference, wing length, generation time

## Abstract

Trait data are fundamental for many aspects of ecological research, particularly for modeling species response to environmental change. We synthesised information from the literature (mainly field guides) and direct measurements from museum specimens, providing a comprehensive dataset of 26 attributes, covering the 43 resident species of Odonata in Britain. Traits included in this database range from morphological traits (e.g. body length) to attributes based on the distribution of the species (e.g. climatic restriction). We measured 11 morphometric traits from five adult males and five adult females per species. Using digital callipers, these measurements were taken from dry museum specimens, all of which were wild caught individuals. Repeated measures were also taken to estimate measurement error. The trait data are stored in an online repository (https://github.com/BiologicalRecordsCentre/Odonata_traits), alongside R code designed to give an overview of the morphometric data, and to combine the morphometric data to the single value per trait per species data.

## Introduction

Trait data are vital components of many ecological, evolutionary and conservation research projects. A useful definition for a trait was provided by Violle et al. (2007): “Any morphological, physiological or phenological feature measurable at the individual level, from cell to whole-organism level, without reference to the environment or any other level of organisation”. Additionally, traits (or attributes) based on species distributions, such as range size or realised climatic niche can also be estimated. The trait characteristics of a species reflect its evolutionary adaptation to certain biotic and abiotic conditions, for example the fur of polar bears (*Ursus maritimus*) is vital for retaining warmth and for camouflage when hunting. Traits are frequently used to categorise groups of species, an approach that has been used to a great effect in the expanding field of functional diversity research (Petchey and Gaston 2006). Here species are grouped by traits that are believed to impact either directly or indirectly on species fitness (Violle et al. 2007). This approach has been extended further by linking traits to certain ecosystem services (de-Bello et al. 2010). The comparative analysis literature is another area of research where trait data play an important role. Here traits are used to understand variation in certain response variables across multiple species (Fisher and Owens 2004; Díaz et al. 2013). For example, comparative trait-based studies are often used to examine whether certain character traits predispose species to distributional change during times of environmental change (Purvis et al. 2000; Reynolds et al. 2005; Walker and Preston 2006; Angert et al. 2011; Grewe et al. 2013). An advantage of such trait-based approaches is that they can allow generalisations to be made across multiple species and can help infer the key drivers of change (Fisher and Owens 2004; Koh et al. 2004; Cardillo et al. 2005). The rise in public participation in biological recording, and the resulting increase in distribution data, has enabled trends in distributions to be estimated for a wide number of taxonomic groups (Silvertown 2009; Dickinson et al. 2012; Burns et al. 2013). Comparative trait-based analysis is an ideal approach for interpreting these distribution trends, however such analyses are currently limited by a lack of trait data.

There are 43 resident species of Odonata in Britain (dependent on taxonomic classification), including widespread common species and rarer species with a restricted range. Odonata have shown phenological and distributional shifts in response to climate change, and habitat destruction has caused widespread declines across many species (Hickling et al. 2005; Brooks and Lewington 2007; Hassall and Thompson 2008). They are beneficial as they feed on many insect pests (Brooks and Lewington 2007), and their sensitivity to the degradation of natural water ecosystems mean they are useful bioindicators (Samways and Steytler 1996; Sahlén and Ekestubbe 2001; Foote and Hornung 2005). Odonata are also among the most charismatic insect groups, which has generated large quantities of data and makes dragonflies an important group for trait-based comparative research. Here we synthesise data from the literature, predominantly field guides, and direct measurements from museum specimens to provide a comprehensive trait database of the Odonata in Britain.

The trait data we present are applicable to a wide range of research projects, studies aimed at understanding and predicting the dynamics of Odonata during times of environmental change will particularly benefit from this data.

## Sampling methods

### Study extent

We provide a comprehensive trait database covering 26 attributes of the 43 resident species of Odonata found in Britain. The database consists of two sections: 1) trait data with a single trait value per species and 2) a database of multiple measurements per species for 11 morphological traits. The data are stored in an online repository (https://github.com/BiologicalRecordsCentre/Odonata_traits), alongside R code designed to combine the two datasets and to give an overview of the available trait data.

### Sampling description

*Single value per species data*

The single value per species dataset contained 15 attributes, these include: *body size*, *body length*, *larval body length*, *generations per year*, *overwintering stage*, *flight period*, *flight power*, *length of activity period*, *migratory*, *distribution status*, *JNCC Red List status*, *synchronicity*, *climate restrictions*, *oviposition*, *life cycle*, *breeding habit* and *various habitat metrics*. A variety of sources and expert opinion were used in the collation of the single trait per species trait dataset. An explanation of each trait can be found in the *Key* tab of *Trait_data_and_sources.xls* file in the *data* folder of the GitHub repository. This .xls file also contains all information regarding the original sources of the data. It should be noted that traits derived from distribution data, for example *distribution status and climate restrictions*, are subject to the quality of the distribution data and should therefore be interpreted with caution. The polygon distribution maps commonly found within field guides are simplifeid coarse-scale interpretations of a species' extent of occurence. We believe that the these coarse-scale range maps are suitable given the broad categories in which species are grouped for the two distribution based traits in this database (*distribution status and climate restrictions*).

*Morphological trait measurement data*

We measured several morphological trait variables from dry mounted specimens of Odonata held at the Natural History Museum of London. Specimens were selected based on a pair of random numbers, one for the draw number and the other for specimen number. Specimens could only be selected if they were complete and undamaged. Measurements were made using digital callipers on wild caught adult specimens. Traits measured were: the length, width and depth of the thorax, and the length and width of all four wings (all in mm, with a precision of 0.1 mm). Each trait was measured for 10 individuals from each species, 5 males and 5 females.

*R code*

**Figure 1.**
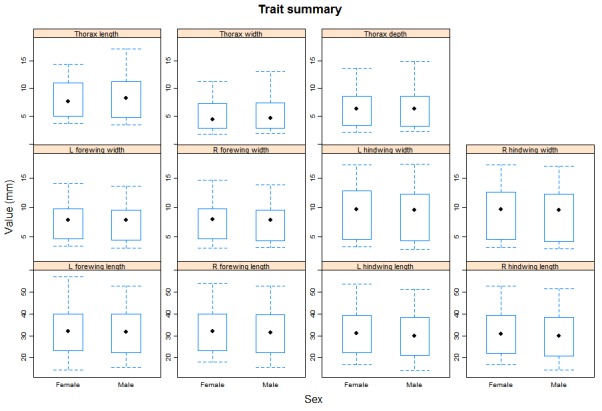
A graphical summary of the morphological trait data across all species. Each trait is represented by a seperate box and whisker plot, which in turn is seperated by sex.

**Figure 2.**
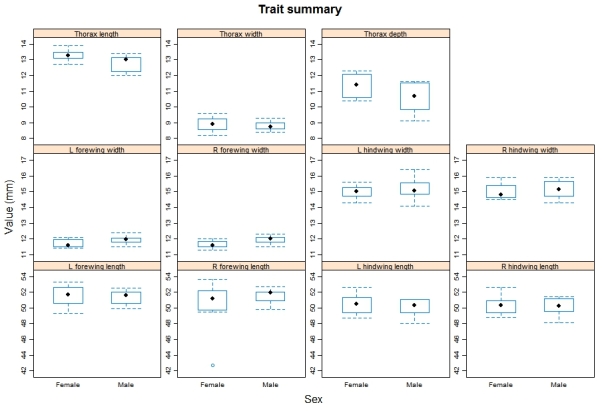
A graphical summary of the morphological trait data collected for *Anax imperator*. Each trait is represented by a seperate box and whisker plot, which in turn is seperated by sex.

We provide two R scripts alongside the trait data in the online repository (https://github.com/BiologicalRecordsCentre/Odonata_traits/tree/master/Scripts). The first, *Odonata_trait_summary.r*, provides an overview of the morphological trait measurement data. Within the code we have written the function, *trait_summary*, which produces a series of boxplots highlighting the median and spread of the measurement data for each trait split by sex (See Figs 1, 2 for examples). The second, *Combine_odonata_trait_data.r*, identifies the mean value across all measurements per trait, per species, and then merges this with the single value per species dataset.

### Quality control

Repeat measures of each trait were taken from one specimen per species, *repeated_trait_measurements.csv.* These were used to estimate trait measurement error, which we found were accurate to an average of 0.12 mm.

### Step description

The single value per trait per species dataset was derived from a literature search (Hammond 1983; d'Aguilar et al. 1986; Shirt 1987; Merritt et al. 1996; Powell 1999; Dijkstra and Lewington 2006; Corbet and Brooks 2008; Brooks and Lewington 2007; Cham 2007; Corbet and Brooks 2008; Daguet et al. 2008; Cham 2009; Smallshire and Swash 2010; Corbet et al. 2013), while direct measurements from museum specimens were taken to compile the morphometric data. The data are stored in an online repository (https://github.com/BiologicalRecordsCentre/Odonata_traits), alongside R code designed to combine the two datasets and to give an overview of the available trait data.

## Geographic coverage

### Description

Data were collated for the 43 resident species of Odonata in Britain.

### Coordinates

-90 and 59.00 Latitude; -11.00 and 2.00 Longitude.

## Taxonomic coverage

### Description

We provide trait data for the 43 resident species of Odonata in Britain.

### Taxa included

**Table:** 

Rank	Scientific Name	Common Name
species	*Aeshna affinis*	Southern Migrant Hawker
species	*Aeshna caerulea*	Azure Hawker
species	*Aeshna cyanea*	Southern Hawker
species	*Aeshna grandis*	Brown Hawker
species	*Aeshna isosceles*	Norfolk Hawker
species	*Aeshna juncea*	Common Hawker
species	*Aeshna mixta*	Migrant Hawker
species	*Anax imperator*	Emperor Dragonfly
species	*Brachytron pratense*	Hairy Dragonfly
species	*Calopteryx splendens*	Banded Demoiselle
species	*Calopteryx virgo*	Beautiful Demoiselle
species	*Ceriagrion tenellum*	Small Red Damselfly
species	*Coenagrion hastulatum*	Northern Damselfly
species	*Coenagrion lunulatum*	Irish Damselfly
species	*Coenagrion mercuriale*	Southern Damselfly
species	*Coenagrion puella*	Azure Damsefly
species	*Coenagrion pulchellum*	Variable Damselfly
species	*Coenagrion scitulum*	Norfolk Damselfly
species	*Cordulegaster boltonii*	Golden-ringed Dragonfly
species	*Cordulia aenea*	Downy Emerald
species	*Enallagma cyathigerum*	Common Blue Damselfly
species	*Erythromma najas*	Red-eyed Damselfly
species	*Erythromma viridulum*	Small Red-eyed Damselfly
species	*Gomphus vulgatissimus*	Club-tailed dragonfly
species	*Ischnura elegans*	Blue-tailed Damselfly
species	*Ischnura pumilio*	Scarce Blue-tailed Damselfly
species	*Lestes barbarus*	Southern Emerald Damselfly
species	*Lestes dryas*	Scarce Emerald Damselfly
species	*Lestes sponsa*	Emerald Damselfly
species	*Lestes viridis*	Willow Emerald Damselfly
species	*Leucorrhinia dubia*	White-faced Darter
species	*Libellula depressa*	Broad-bodied Chaser
species	*Libellula fulva*	Scarce Chaser
species	*Libellula quadrimaculata*	Four-spotted Chaser
species	*Orthetrum cancellatum*	Black-tailed Skimmer
species	*Orthetrum coerulescens*	Keeled Skimmer
species	*Platycnemis pennipes*	White-legged Damselfly
species	*Pyrrhosoma nymphula*	Large Red Damselfly
species	*Somatochlora arctica*	Northern Emerald
species	*Somatochlora metallica*	Brillinat Emerald
species	*Sympetrum danae*	Black Darter
species	*Sympetrum sanguineum*	Ruddy Darter
species	*Sympetrum striolatum*	Common Darter

## Traits coverage

We synthesised information from the literature (mainly field guides) and direct measurements from museum specimens, providing a comprehensive dataset of 26 attributes, covering the 43 resident species of Odonata in Britain. Traits included in this database range from morphological traits (e.g. body length) to attributes based on the distribution of the species (e.g. climatic restriction). A detailed description of the trait data can be found in the data resources section below.

## Usage rights

### Use license

Creative Commons CCZero

## Data resources

### Data package title

Odonata traits

### Resource link

https://github.com/BiologicalRecordsCentre/Odonata_traits

### Number of data sets

4

### Data set 1.

#### Data set name

Species trait measurements

#### Data format

.csv

#### Number of columns

18

#### Description

**Data set 1.** 

Column label	Column description
Specimen_Number	Unique identifier for the specimen (based on the specimen number in Natural History Museum, London)
Preservation	Preservation method of the specimen
Order	The species taxonomic order
Family	The species taxonomic family
Genus	The species taxonomic genus
Species	The species name
Sex	The sex of the specimen
thorax_length	The length of the thorax in mm
thorax_width	The width of the thorax (widest point) in mm
thorax_depth	The depth of the thorax in mm
left_forewing_length	The length of the left forewing in mm
left_forewing_width	The width of the left forewing (at the widest point) in mm
left_hindwing_length	The length of the left hindwing in mm
left_hindwing_width	The width of the left hindwing (at the widest point) in mm
right_forewing_length	The length of the right forewing in mm
right_forewing_width	The width of the right forewing (at the widest point) in mm
right_hindwing_length	The length of the right hindwing in mm
right_hindwing_width	The width of the right hindwing (at the widest point) in mm

### Data set 2.

#### Data set name

Odonata traits single

#### Data format

.csv

#### Number of columns

33

#### Description

**Data set 2.** 

Column label	Column description
Species	Species name
Suborder	The suborder of the species
Family	The family of the species
Body_size_min	The minimum length of the abdomen (mm)
Body_size_max	The maximum length of the abdomen (mm)
Body_size_median	The median length of the abdomen (mm)
Partivoltine	A series of voltinism types (Partivoltine, Semivoltine, Univoltine, Bivoltine, Multivoltine), listed in increasing order of voltinism. Species are given a 1 if they express that level of voltinism in the UK, whereas 1* is used to show species that have expressed this level of voltinism outside of the UK.
Semivoltine	A series of voltinism types (Partivoltine, Semivoltine, Univoltine, Bivoltine, Multivoltine), listed in increasing order of voltinism. Species are given a 1 if they express that level of voltinism in the UK, whereas 1* is used to show species that have expressed this level of voltinism outside of the UK.
Univoltine	A series of voltinism types (Partivoltine, Semivoltine, Univoltine, Bivoltine, Multivoltine), listed in increasing order of voltinism. Species are given a 1 if they express that level of voltinism in the UK, whereas 1* is used to show species that have expressed this level of voltinism outside of the UK.
Bivoltine	A series of voltinism types (Partivoltine, Semivoltine, Univoltine, Bivoltine, Multivoltine), listed in increasing order of voltinism. Species are given a 1 if they express that level of voltinism in the UK, whereas 1* is used to show species that have expressed this level of voltinism outside of the UK.
Multivoltine	A series of voltinism types (Partivoltine, Semivoltine, Univoltine, Bivoltine, Multivoltine), listed in increasing order of voltinism. Species are given a 1 if they express that level of voltinism in the UK, whereas 1* is used to show species that have expressed this level of voltinism outside of the UK.
Overwintering_stage	Categorical variable with 3 levels: Larvae (L), Eggs (E), Eggs & Larvae (EL)
Flight_period_start	The start (month) of the flight period.
Flight_period_end	The end (month) of the flight period.
Flight_period_duration	The duration of the flight period in months.
Migratory	Binary variable, do adults routinely migrate to/from the UK.
Distribution_status	Categorical variable that groups species based on distribution size: very widespread, widespread, local, scarce, rare, very rare.
JNCC_Red_List_status	Species classified using the national Red List criteria.
Synchronicity	A variable describing a species emergence strategy: 1) Spring emergence = highly synchronised, 2) Summer emergence = temporally dispersed emergence, 3) Type 3 Summer = Obligatorily univoltine species.
Climatic_restrictions	Broad climatic categorisation of species based on their distribution pattern: 1) Widespread, 2) Southern, 3) Northern, 4) Continental, 5) Oceanic.
Lowland_rivers_and_canals	A series of habitat types, species are given a 1 if they utilise the habitat in question.
Streams_and_upland_rivers	A series of habitat types, species are given a 1 if they utilise the habitat in question.
Bogs_moorland_and_lowland_wet_heath	A series of habitat types, species are given a 1 if they utilise the habitat in question.
Levels_fens_and_grazing_marshes	A series of habitat types, species are given a 1 if they utilise the habitat in question.
Ponds_and_lakes	A series of habitat types, species are given a 1 if they utilise the habitat in question.
Woodland	A series of habitat types, species are given a 1 if they utilise the habitat in question.
No_habitat_types	A count of the number of habitat types utilised by the species
Oviposition	The oviposition strategy of the species, binary variable where species are classified as either Endophytic or Exophytic
breeding_habitat	Species are classified into groups based on their preferred breeding habitat, either lentic or lotic. Species that are able to utilise both types are listed as lentic/lotic.
Body_length_min	The minimum value for total body length (mm)
Body_length_max	The maximum value for total body length (mm)
Larval_body_length_min	The minimum value for total length of the larvae (mm)
Larval_body_length_max	The maximum value for total length of the larvae (mm)

### Data set 3.

#### Data set name

Trait data and sources

#### Data format

.xls

#### Number of columns

3

#### Description

This .xls file contains three sheets. The first lists the same traits that are listed in the "Odonata traits single" .csv, but with additional columns after each trait that refer the reader to the unique identifier of source of the data. The second sheet contains a list of sources with their unique identifiers. The third sheet contains a key to the traits that are included in sheet 1.

**Data set 3.** 

Column label	Column description
Sheet 1: Data	Contains the traits that are listed in the "Odonata traits single" .csv, but with additional columns after each trait that refer the reader to the unique identifier of source of the data
Sheet 2: Sources	Contains a list of sources with their unique identifiers
Sheet 3: Trait key	Contains a key to the traits that are included in sheet 1

### Data set 4.

#### Data set name

Repeated trait measurements

#### Data format

.csv

#### Number of columns

12

#### Description

**Data set 4.** 

Column label	Column description
Specimen_Number	Unique identifier for the specimen (based on the specimen number in Natural History Museum, London)
thorax_length	The length of the thorax in mm
thorax_width	The width of the thorax (widest point) in mm
thorax_depth	The depth of the thorax in mm
left_forewing_length	The length of the left forewing in mm
left_forewing_width	The width of the left forewing in mm
left_hindwing_length	The length of the left hindwing in mm
left_hindwing_width	The width of the left hindwing in mm
right_forewing_length	The length of the right forewing in mm
right_forewing_width	The width of the right forewing in mm
right_hindwing_length	The length of the right hindwing in mm
right_hindwing_width	The width of the right hindwing in mm
